# Detection and Characterization of Nodularin by Using Label-Free Surface-Enhanced Spectroscopic Techniques

**DOI:** 10.3390/ijms232415741

**Published:** 2022-12-12

**Authors:** Ioana Andreea Brezeștean, Ana Maria Raluca Gherman, Alia Colniță, Nicoleta Elena Dina, Csilla Müller Molnár, Daniel Marconi, Vasile Chiș, Ioan-Leontin David, Simona Cîntă-Pînzaru

**Affiliations:** 1 Department of Molecular and Biomolecular Physics, National Institute for Research and Development of Isotopic and Molecular Technologies, 400293 Cluj-Napoca, Romania; 2 Faculty of Physics, Babeş-Bolyai University, 400084 Cluj-Napoca, Romania

**Keywords:** Raman, SERS, nodularin, cyanotoxin, microcystins

## Abstract

Nodularin (NOD) is a potent toxin produced by *Nodularia spumigena* cyanobacteria. Usually, NOD co-exists with other microcystins in environmental waters, a class of cyanotoxins secreted by certain cyanobacteria species, which makes identification difficult in the case of mixed toxins. Herein we report a complete theoretical DFT-vibrational Raman characterization of NOD along with the experimental drop-coating deposition Raman (DCDR) technique. In addition, we used the vibrational characterization to probe SERS analysis of NOD using colloidal silver nanoparticles (AgNPs), commercial nanopatterned substrates with periodic inverted pyramids (Klarite^TM^ substrate), hydrophobic Tienta^®^ SpecTrim^TM^ slides, and in-house fabricated periodic nanotrenches by nanoimprint lithography (NIL). The 532 nm excitation source provided more well-defined bands even at LOD levels, as well as the best performance in terms of SERS intensity. This was reflected by the results obtained with the Klarite^TM^ substrate and the silver-based colloidal system, which were the most promising detection approaches, providing the lowest limits of detection. A detection limit of 8.4 × 10^−8^ M was achieved for NOD in solution by using AgNPs. Theoretical computation of the complex vibrational modes of NOD was used for the first time to unambiguously assign all the specific vibrational Raman bands.

## 1. Introduction

Cyanobacteria, also known as blue-green algae, although strictly speaking are not algae, represent up to 70% of the entire phytoplankton biomass and are thought to be the first oxygenic photosynthetic microorganisms on Earth [1,2,3]. They populate almost every freshwater and marine environment and use sunlight as an energy source to produce biomass from carbon dioxide (CO_2_). Without effective mitigation strategies for their overgrowth in eutrophic conditions, massive blooms of cyanobacteria can seriously affect water quality, by inducing toxicity in freshwater and marine environments [2]. Moreover, cyanobacterial blooms can induce liver, digestive, and neurological diseases through ingestion by living organisms [4,5,6,7] and biological incorporation into spray aerosol [8]. Thus, toxin-producing cyanobacteria are recognized as a threat to public health globally, particularly in regions without access to high-quality drinking water.

During flowering, cyanobacteria produce strong toxins, such as hepatotoxic microcystins (MCs) and nodularins (NODs) [9,10,11,12]. MCs and NODs are hepta- and pentapeptides with very similar structures, which have been shown to cause hepatotoxicity by inhibiting protein phosphate 1A and 2A (PP1 and PP2A) that lead to the intensive use of vital cellular proteins. Both toxins are recognized as potential tumor promoters and carcinogens, and hence it is of utmost importance to trace their presence in the environment in order to eliminate even a low-level exposure to humans [13,14] through seafood, such as mussels, shellfish and fish [15]. After their ingestion, these toxins are absorbed from the ileum into the bloodstream and processed by the liver through multispecific transmembrane organic anion transporters [16].

NODs are produced by the filamentous cyanobacterium *Nodularia spumigena* [17] and benthic species *Nodularia sphaerocarpa* PCC7804 [18,19,20]. Their biosynthesis is regulated by genes and performed non-ribosomally according to a similar mechanism involved in MC production [21]. Currently, ten structural variants of NODs are known [22]. These types of protein-bound MCs have not been detected using well-known analytical approaches; thus, the actual content of MCs in cyanobacterial blooms has been underestimated [23]. Due to the important health risks that these MCs and NODs present to living organisms, safe and accurate trace-level detection techniques are much needed. These methods should be highly sensitive, fast and reliable, and capable of detecting multiple MS variants generated and in low concentration [24].

As recently reviewed [25] the current analytical methods used to detect MCs are biological (mouse bioassay—MBA), biochemical (protein phosphatase inhibition assay—PPIA, and enzyme-linked immunosorbent assay—ELISA), and chemical (high-performance liquid chromatography—HPLC, liquid chromatography-mass spectrometry—LC-MS, high-performance capillary electrophoresis—HPCE, and gas chromatography—GC), as well as newly emerging biosensing methods [25].

MBA is a realistic, qualitative approach for detecting a specific MC variant and toxicity in the whole animal [26,27]. It has serious limitations, such as, a lack of sensitivity, being inappropriate for quantification purposes or large-scale and routine testing, and requiring a large number of mice and a license in order to be performed [28,29].

The PPIA method is very appropriate, as MCs are specific inhibitors of PP1 and PP2A [30,31,32]. It is relatively cheap, simple, fast, and sensitive enough to detect and quantify MCs in water below a 1 μg·L^−1^ threshold, as proposed by WHO’s drinking-water guidelines. However, it does not provide information on the toxicity of MC variant(s) and has no specificity for these [33,34,35]. Recently, Wharton et al. [36] reported the incorporation of an immunocapture protein phosphatase inhibition assay to improve the sensitivity and specificity of traditional PPIA techniques for monitoring low-level human exposure to MCs and NODs.

Although ELISA meets all the requirements for a low-sensitivity screening technique, it is hampered by high equipment costs, long analysis time, and the need for trained personnel [37,38,39,40]. The high-throughput approaches for cyanotoxin analysis providing quantification and multiple-source detection still rely on LC-based methods coupled to MS, which involve complex sample pretreatment.

**Figure 1:**
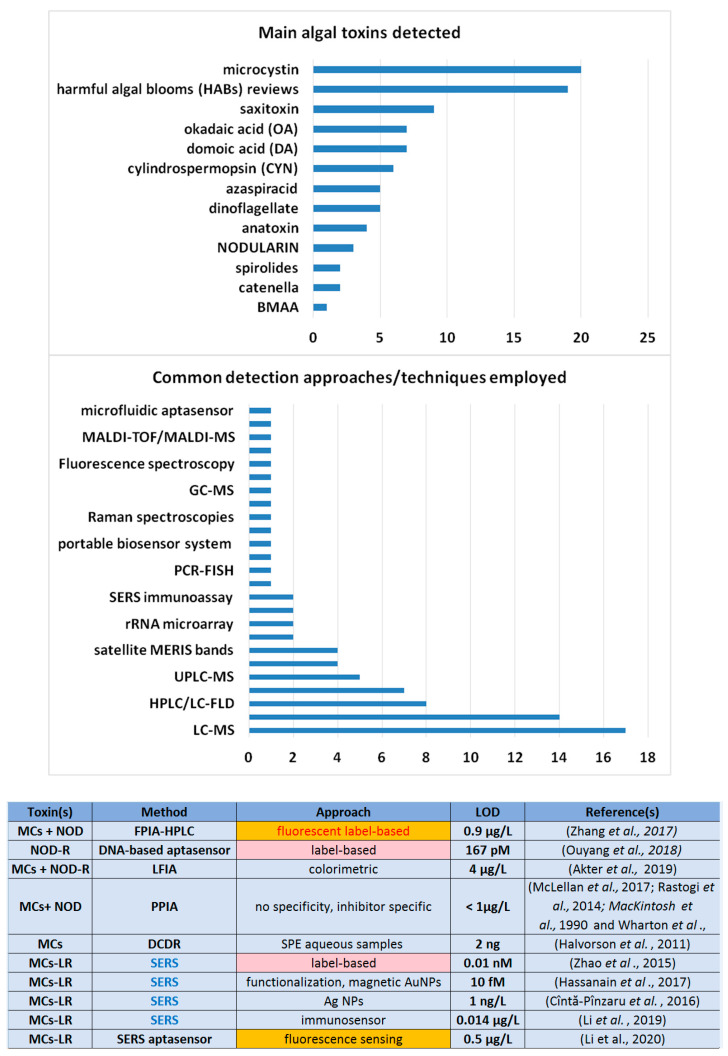
Main algal toxins correlated with blooming, and the most relevant techniques used for their detection as reported between 2017 and 2022 (source—Web of Science Collection). A table summarizing the lowest LODs obtained by using label-based approaches or elaborated platforms designed for trace-level detection [30,31,32,36,48,49,50,54,57,63,64,65,67].

A survey over last 5 years that includes 150 research articles and reviews on harmful algal blooms (HABs) and their detection (Figure 1) revealed that microcystins are of the most interest along with several particular toxins from the paralytic shellfish poisoning (PSP), diarrhetic shellfish poisoning (DSP), amnesic shellfish poisoning (ASP) and azaspiradids (AZA) groups, such as saxitoxin (STX), okadaic acid (OA), domoic acid (DA), and azaspiracid, respectively [41,42,43].

Among the methods used for their ultrasensitive detection, the most employed is LC-MS or MS in combination with GC, UPLC—ultra-performance liquid-chromatography tandem-mass spectrometry (UPLC-MS/MS) or with MALDI-TOF. Several other modern techniques were successfully used for fish-killing toxins’ trace-level detection: LFD (lateral flow dipstick); ELISA; self-assembled monolayer (SAM)-based immunoassays; recombinase polymerase amplification (RPA); spatiotemporal distribution by solid-phase adsorption toxin tracking (SPATT); high-speed microscale imaging system (HSMIS); nuclear-based radioligand-receptor binding assay (RBA); time-resolved fluorescence immunoassay (TRFIA), etc.

Common eider (*Somateria mollissima*) liver samples collected from 15 birds shot in the northern Baltic Sea were used by Sipia et al. [44] to document for the first time the presence of NOD in seabirds and NOD transfer in the Baltic Sea food web with the aid of a combination of ELISA and LC-MS methods. Using the same methods, the group also examined NOD bioaccumulation in northern Baltic Sea flounder livers [45]. In September 2002, maximum toxin concentrations up to 390 μg/kg dw were found using LC-MS and up to 2230 μg/kg dw using ELISA techniques. They have also detected notable NOD concentrations in liver and muscle samples from common eider, roach, and flounder caught from the northern Baltic Sea demonstrating [46] the need for screening and risk assessment of NOD. A first report of *Nodularia spumigena* blooms in sub-tropical Australia and NOD bioaccumulation in isolated populations of mullet was conducted by Stewart et al. [47]. Moreover, the authors included a systematic review of the literature regarding NOD bioaccumulation in edible fish, shellfish, and crustaceans.

By using HPLC, Zhang et al. [48] developed a new strategy for environmental sample analysis based on fluorescence polarization immunoassay (FPIA) for detecting MCs and NODs in water using fluorescein tracers that were synthesized and purified.

Ouyang et al. [49] reported a NOD-R detection limit as low as 167 pM using a newly developed DNA-based aptasensor with high selectivity and good reproducibility and stability. A more recent study [50] proposes a simple chromogenic lateral-flow immunoassay (LFIA) approach for the simultaneous detection of MC and NOD-R concentrations lower than 4 μg/L. One of the advantages of this reported detection method is the possibility to visually confirm the result without using additional measuring devices.

Raman spectroscopy (RS) is a powerful analytical and nondestructive technique that uses light to identify the unique spectral fingerprint of molecules [51]. The Raman spectra can be collected from aqueous or solid samples deposited on transparent substrates such as plastic or glass. In recent years, RS has become a popular tool to diagnose infectious diseases and detect toxins [41,42,43], due to its high sensitivity to slight changes of low sample concentrations in biological environments [51,52,53]. Pure toxin samples containing down to 2 ng of MC-LR toxin have been successfully identified and quantified using the drop-coating deposition Raman (DCDR) technique [53,54]. Using a μ-RIM™ stainless steel hydrophobic substrate, the NIR-Raman spectrum of okadaic acid, a DSP toxin, has been recorded from 75 µg recrystallized toxin from commercial solution, after drop-coating deposition [42]. Furthermore, using surface enhanced Raman scattering (SERS), 8.3 μg cylindrospermopsin in fish tissue was detected [55].

In comparison to normal Raman spectroscopy, the surface-enhanced Raman scattering (SERS) method exploits the plasmonic properties of the noble metal nanoparticles to exhibit an enhanced Raman signal of the molecule placed in close vicinity to the plasmonic substrate. Thanks to its advantages such as simplicity, minimal sampling protocol, photostability, reliable quantification, and multiplexing capability, SERS has emerged as a powerful analytical tool in biomedical applications [56,57,58,59,60,61]. SERS-based biosensors for MC detection have proven to be fast, highly sensitive, non-destructive, and easy-to-use. They integrate the molecular-specific Raman fingerprinting of MCs with the potential for single-molecule sensitivity [62]. MC-LR detection of trace levels down to 0.01 nM has been achieved using labeled SERS technology [63], while functionalized gold-coated magnetic nanoparticles (NPs) have been used for the selective capture of traces of MC-LR in complex water bodies [64]. Moreover, traces of MC-LR have been characterized using silver (Ag) as a SERS substrate [65]. Colloidal AgNPs were often employed to characterize certain marine microorganisms and their secretion of extracellular polymeric matrix [66]. Recently, a new SERS immunosensor for the detection and quantification of MC-LR toxin in aquatic settings has been reported [57] with a detection limit of 0.014 μg/L, while the same group later developed a sensitive and selective aptasensor for fluorescence−SERS dual-modal detection of MC-LR toxins [67]. Luo et al. [68] fabricated a planar silicon aptasensor constructed from successive layers of gold (Au) core–SERS label–Ag shell–Au shell and functionalized on the outer Au surface by MC-LR and/or MC-RR aptamers that can indirectly detect MC-LR and MC-RR, individually or simultaneously, in natural water and in algal culture. A recently published review explores the current state of the art of aptasensor-based platforms and their limitations for the most efficient detection of cyanobacteria associated toxins [69].

To correctly detect cyanotoxins in various designed sensing schemes relying on Raman spectroscopy (or SERS), a comprehensive vibrational Raman spectroscopy description of the targeted compound is needed. Limited information regarding Raman spectroscopy of nodularin is available [53,54]. Halvorson et al. [53] analyzed NOD as part of a DCDR Raman study to classify 72 DCDR spectra belonging to the eight toxins, seven microcystins variants and nodularin, respectively. Since Raman spectra reported in the range 500–1800 cm^−1^ appeared rather similar, the authors proposed toxin bands which could help discriminate between microcystin variants. They used two approaches, one relying on typical intensity ratio of bands attributable to amino acids to discriminate the eight toxins according to their specific functional groups, as well as a PCR-discrimination approach. Doing so, Halvorson et al. reported only experimental NOD Raman spectra in the 500–1800 cm^−1^ range, using a 785 nm laser line for excitation. There are no studies regarding the limit of detection (LOD) in solutions using Raman or SERS. Neither theoretical computation of the complex vibrational modes of NOD can unambiguously assign all the vibrational Raman bands observed in the whole spectral range (100–3600 cm^−1^). Thus, here we report the first complete experimental Raman and computational DFT study of nodularin in solutions or adsorbed on various SERS substrates.

For DCDR analysis several approaches were used to improve the limit of detection. We employed the commercially available hydrophobic slides Tienta^®^ SpectRIM^TM^ to reduce the DCDR limit of detection to 10^−3^ M. By using a commercial nanopatterned substrate consisting of periodic pyramidal square pits (Klarite™), a SERS limit of detection was further reduced (10^−7^ M). In-house, fabricated by NIL, periodic nanotrenches, covered with a nanostructured silver (Ag) film, 25 nm thick, deposited by direct current (DC) sputtering were also tested. The SERS results were compared with those obtained using the classical, citrate-reduced colloidal AgNPs. Thus, by starting from several previous studies as a fundamental basis of our work, we report on a comprehensive characterization of NOD by using label-free surface-enhanced spectroscopic techniques.

## 2. Results and Discussion

### 2.1. Raman Analysis of NOD

**Figure 2:**
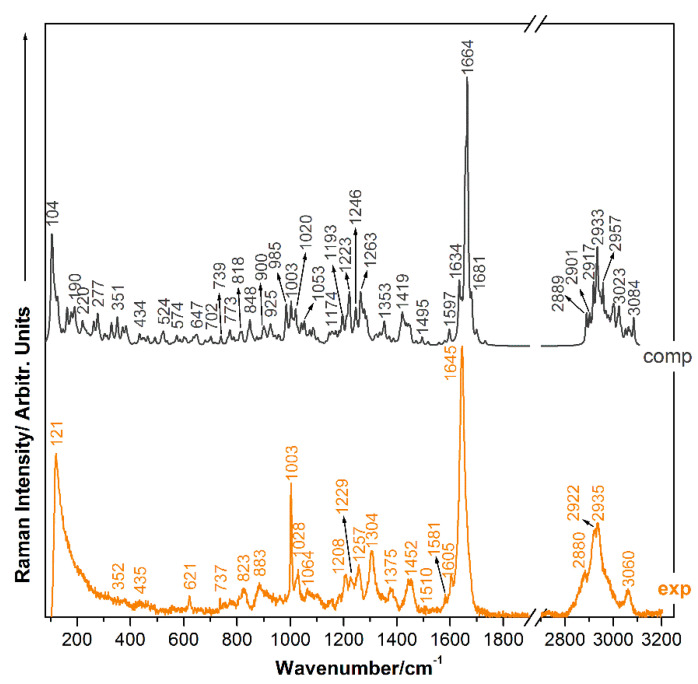
Raman spectrum of NOD (grey) calculated at AFPD/6-311+G(2d,p) level of theory as compared to experimental spectrum (orange) registered with 633 nm laser line under ambient conditions.

The Raman spectrum of NOD registered by using a 633 nm excitation laser line is plotted in Figure 2 as compared to its theoretical correspondent calculated at harmonic level. The Raman spectra were acquired from a solid sample, recrystallized from a solution of 10^−3^ M NOD (by dissolving 100 µg in 100 µL of ethanol). The reason for selecting several experimental approaches was that by using only DCDR we could not determine with high accuracy the final concentration of the sample. Signal acquisition revealed higher intensity when analyzing the outer edge of the coffee-ring-shaped droplet dried on a hydrophobic surface. Therefore, by examining separate regions from the droplet, the Raman signal can fluctuate. Moreover, in real aquatic environmental samples, NOD is present diluted in complex matrices containing carotenoids, minerals, and biomass [66], so analyzing solutions containing trace levels of NOD is highly relevant for further applications.

As demonstrated in previous studies [70,71,72], DCDR analysis showed detection sensitivity superior to conventional Raman for small biological molecules, such as acetylsalicylic acid, riboflavin, and contaminants [73,74], down to a detection limit of 10^−8^ M. It also proved to be an important tool for membrane-interaction studies, such as the liposome–porphyrin complex [75], for the quantitative determination of creatinine in urine [76], and for colorectal cancer detection in blood plasma [77]. The advantages of DCDR analysis include the use of dried, preconcentrated samples, a small sample volume, no interference from solvents, and the capability to segregate any existing impurities [76]. Therefore, when the signal acquisition takes place from the outer edge of the coffee ring-shaped droplet dried on a hydrophobic surface, a significantly improved Raman signal is obtained.

During the DCD process, when an aqueous droplet containing an analyte of interest is dried on various substrates, a possible residual reaction is expected after evaporation of the solvent, which shows spatial variability. The NOD cyanotoxin sample was selected for analysis due to the lack of comprehensive spectral studies specifically for this type of toxin. Only similar toxins called MCs were previously reported in the literature [54].

The strong Raman bands are attributed to the phenylalanine amino acid-**Phe**. For the 1003 cm^−1^, the strongest band in the region of low wavenumbers, we obtained the calculated correspondent with 100 % accuracy, at 1003 cm^−1^, but the band’s intensity is not as well resolved as its position; the very weak bands at 883 cm^−1^ and 823 cm^−1^ are due to a combination of CC stretching and CH_2_ rocking. These two Raman bands are very well resolved in the theoretical spectrum, at 900 cm^−1^ and 818 cm^−1^, respectively. Lastly, the weak splitting at 1208 cm^−1^ is also due to an in-plane deformation of the phenyl ring, calculated at 1020 cm^−1^. In-plane deformation of the phenyl ring coupled with out-of-plane bending of O55H gives a very weak but sharp signal at 621 cm^−1^. 

For the **Adda** group, the bands common to all microcystins are 1208 cm^−1^, 1304 cm^−1^, 1375 cm^−1^, 1452 cm^−1^, and 1645 cm^−1^, as was also shown by Halvorson et al. [54]. From the calculations, we assigned CC and CH stretching moieties combined with in-plane bending of CH groups to the weak but rather sharp band at 1304 cm^−1^. It comes with two calculated correspondents, as shown in Table 1. In- and out-of-phase bending of C41H3-group vibration modes are present in the Raman spectrum of NOD as small bands at 1375 cm^−1^ and 1452 cm^−1^.

The Raman bands observed at 1645 cm^−1^ are characteristic of Amide I and are assigned to the phenyl ring in the **Adda** residue. Particularly, this band has contributions from C=C stretching throughout the whole molecule. Its calculated correspondent is a group of several normal modes, with 1664 cm^−1^ being the strongest, joined by three other shoulders—1681 cm^−1^, 1700 cm^−1^, and 1732 cm^−1^. The latter are not particularly well-resolved in the experimental spectrum. The weak lateral band at 1605 cm^−1^ also corresponds to a C=C stretching. Other present marker bands for the NOD molecule and previously reported in the literature are found in the range of 1200–1300 cm^−1^ and are characteristic to Amide III. The 1208 cm^−1^, 1229 cm^−1^, and 1257 cm^−1^ triplet of weak intensity is calculated at 1193 cm^−1^, 1223 cm^−1^, and 1246 cm^−1^. The first two are due to in-plane bending of CH groups, while the latter is due to the stretching of C57-O58. In contrast to previously reported studies [54], the spectral features found in the 2700–3200 cm^−1^ range are also described in this work. We identified intense bands in the range 2880–3060 cm^−1^ as being present due to symmetrical and asymmetrical stretches of CH, CH_2_, and CH_3_ chemical groups. In this case, a detection limit of only 10^−3^ M was reached, so we investigated further substrates with higher detection performance.

We assigned CC stretching and CH_2_/CH_3_ wagging moiety to the very weak and wide band at 1064 cm^−1^. For this band, we obtained two calculated correspondents, at 1043 cm^−1^ and 1053 cm^−1^.

The strong and wide low-lying frequency band is due to the general vibration of the molecule, more specifically, a rocking of the phenyl ring coupled with the bending of the N28C27N29 group. Its theoretical correspondent lies at 104 cm^−1^.

**Figure 3:**
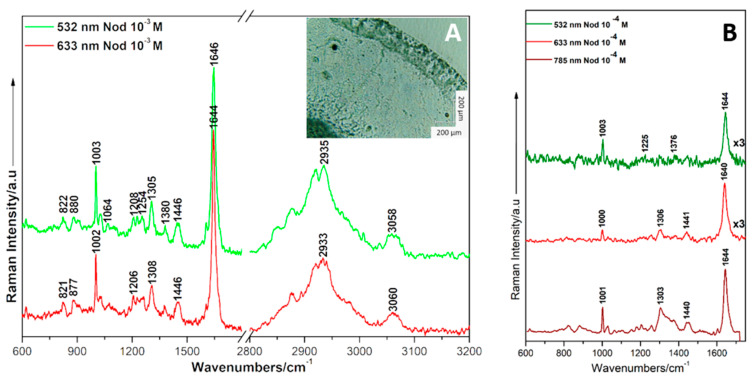
DCDR spectra recorded on Tienta^®^ SpectRIM^TM^ substrate for NOD in ethanol with a final concentration of 10^−3^ M (**A**) and 10^−4^ M (**B**), respectively by using the 785 nm, 532 nm and 633 nm laser lines. Inset with optical image by using 20× objective.

Figure 3A shows the DCD Raman spectra obtained by using different laser lines as excitation sources on a Tienta^®^ SpectRIM^TM^ substrate. The 2 µL sample was deposited and left to air dry, then irradiated for Raman signal acquisition. The marker bands are in good agreement with the reported NOD Raman profile in the literature [54] and show high reproducibility, independent of the laser line.

Figure 3B shows the Raman spectrum of NOD at a concentration of 10^−4^ M. By lowering the concentration, the marker bands of NOD can still be clearly observed at 1003 cm^−1^, 1376 cm^−1^, and the most intense band at 1644 cm^−1^. All the active modes of vibration from the Raman spectrum of NOD registered using a 633 nm excitation laser line as compared to its theoretical correspondent calculated at harmonic level along with their assignments are listed in Table 1.

Furthermore, we tried to obtain a lower detection limit by switching from commercial hydrophobic substrates to the SERS performant substrates. We employed the same drop-coating deposition on top of these substrates and tried to obtain a detection limit below 10^−4^ M.

### 2.2. SERS Analysis of NOD

**Figure 4:**
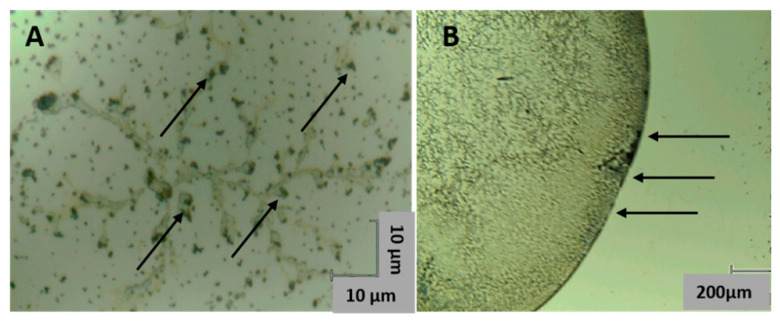

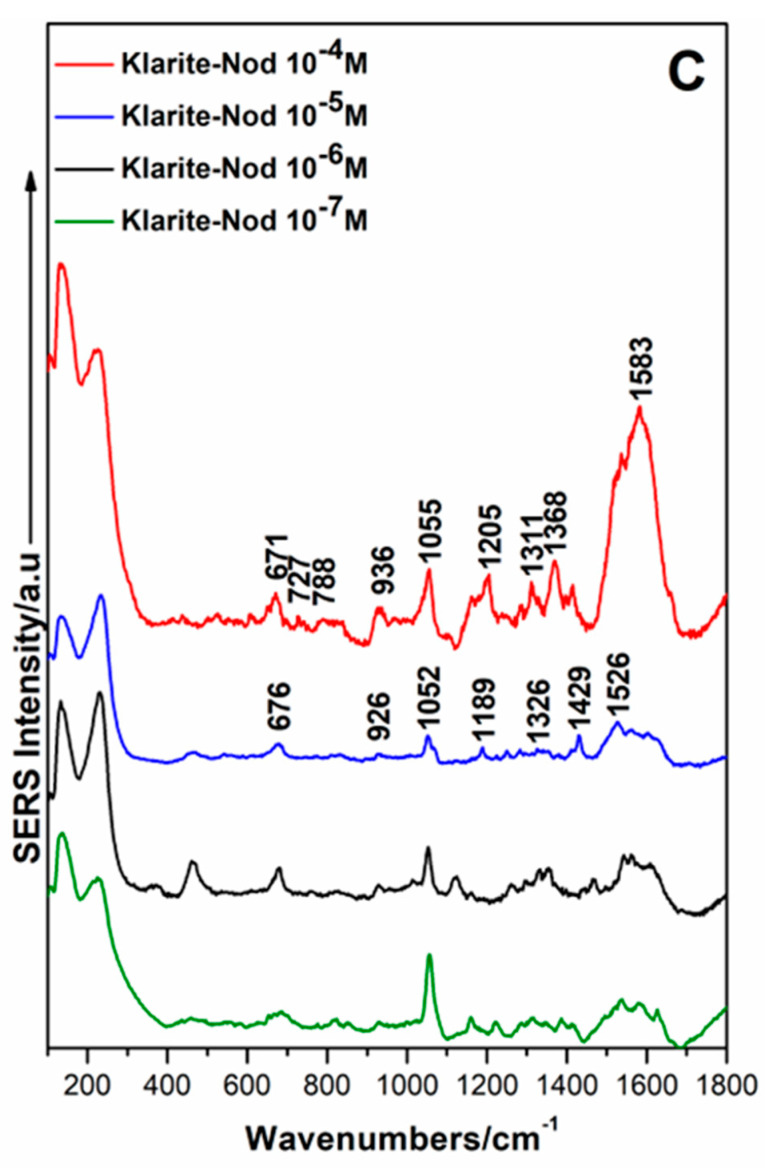
Optical images showing the samples for NOD on Klarite^TM^ substrate by using 100× (**A**) and 20× (**B**) magnification. Arrows show selected points for laser irradiation. SERS spectra recorded on Klarite^TM^ substrate for NOD in ethanol at different concentration using 532 nm laser line (**C**).

In Figure 4A,B we illustrate representative optical images of sample clusters formed after ethanol evaporation. We mainly investigated the “coffee-ring” borderlines, where visible microcrystals of NOD are accumulated; hence, an improved Raman signal is expected. Figure 4C reveals Nodularin’s spectrum on the commercial Klarite^TM^ SERS substrate at different concentrations, as low as 10^−7^ M. Klarite^TM^ substrate was previously used for successful bacteria label-free detection [78], highlighting its SERS performance key aspects such as a strong SERS signal under ambient conditions and the ease of depositing the sample on its surface. 

SERS marker bands of NOD are present in the spectra recorded using the Klarite^TM^ substrate at 1205 cm^−1^, 1311 cm^−1^, 1368 cm^−1^, 1583 cm^−1^ and 1656 cm^−1^, respectively. A medium- to strong-intensity SERS band is present at all concentrations tested at 1055 cm^−1^. This spectral feature is particularly observed in the SERS spectra recorded on the Klarite^TM^ substrate. The periodic inverted pyramids patterned on this substrate might have forced the NOD molecules to accumulate in their cavities in different geometrical configurations and thus, the molecular adsorption could have been realized in several orientations towards the metallic surface. This might influence the SERS spectra by making certain molecular groups more visible in the vibrational fingerprint of the NOD molecule due to their perpendicular orientation to the metallic surface. The bands observed with very strong intensity between 133–234 cm^−1^ support the molecules’ different geometries of adsorption to the metallic surface as revealed also by the DFT calculations (Table 1). The band at 234 cm^−1^ might be a band generally attributed to the chemisorbed atomic-molecular oxygen species, when working in open-air conditions [79]. The 1055 cm^−1^ band is assigned by DFT calculations to the CC stretching vibrations in the C46, C47 and C48 double bonds of the ring. The Raman marker band from 1645 cm^−1^ is very weakly present as a shifted shoulder band at 1656 cm^−1^ in this particular case. 

**Figure 5:**
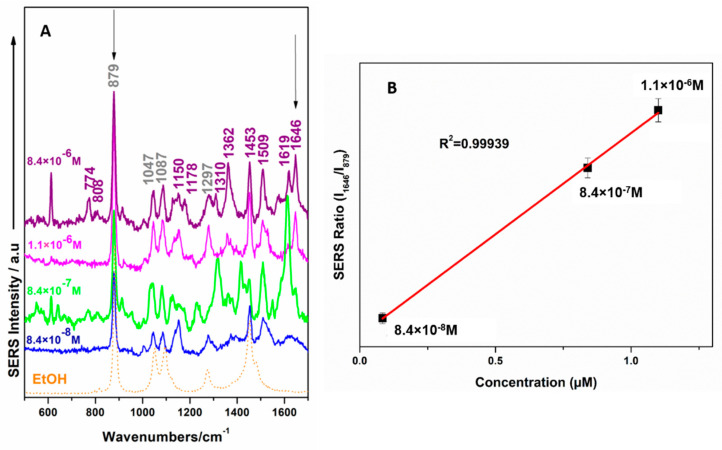
(**A**) SERS spectra on of NOD/ethanol samples at different concentrations using a 532 nm laser line and citrate-reduced AgNPs. (**B**) Linear fit of the relative intensity ratio of the SERS bands at 1647 cm^−1^ and 879 cm^−1^ as a function of NOD concentration. Error bars indicate standard deviation R2 = 0.955.

Figure 5 shows a systematic detection process of NOD by using common Ag sols at different concentrations down to 8.4 × 10^−8^ M. At low concentrations, the SERS spectra also comprise the characteristic bands of ethanol: 878 cm^−1^; 1045 cm^−1^; 1087 cm^−1^; 1453 cm^−1^, respectively (marked in dark grey). The spectral range between 1500 cm^−1^ and 1650 cm^−1^ is significant; the marker band for NOD found at 1647 cm^−1^ becomes increasingly dominant in this region along with the 1509 cm^−1^. The SERS spectral response reflects slight changes from one given concentration value to more reduced ones. Specifically, even though the marker band of NOD found at 1647 cm^−1^ is weak at the lowest detection limit (8.4 × 10^8^ M), the band from 1509 cm^−1^ is intense enough to assure clear identification. The fitting analysis shown in Figure 5B exhibits very good linearity of the relative intensity ratio of the SERS bands found at 1647 cm^−1^ and 879 cm^−1^ as a function of NOD concentration. From the 1.1 × 10^−6^ M value, with the decreasing of the NOD concentration, the intensity ratio of the SERS bands at 1647 cm^−1^ and 879 cm^−1^ are linearly decreased, corresponding to the SERS selection rules [80].

A surprisingly more intense and visible SERS band, as compared to the measurement in a dried droplet on solid substrates, is observed with higher intensity at 1509 cm^−1^ (Figure 5). Moreover, in Raman analysis this band is not significantly present. It could be explained by the possible re-orientation of the molecular structure with respect to the surface in a more tilted position at lower concentrations and a more stand-up orientation of the molecular skeletal ring at higher concentration. Since the vibration found at that spectral position is attributed to the N36H group from the calculated Raman spectrum, we conclude that this group is involved in facilitating adsorption of NOD to the silver surface, most probably through the lone pair of the N atom.

For comparison, since SERS studies on NOD were not found and spectroscopic studies on similar toxins are scarce, we employed an in-house-fabricated SERS solid substrate to exploit its detection potential and provide a full SERS profile of NOD obtained under different experimental conditions. This SERS substrate was previously characterized in our group research [81].

**Figure 6:**
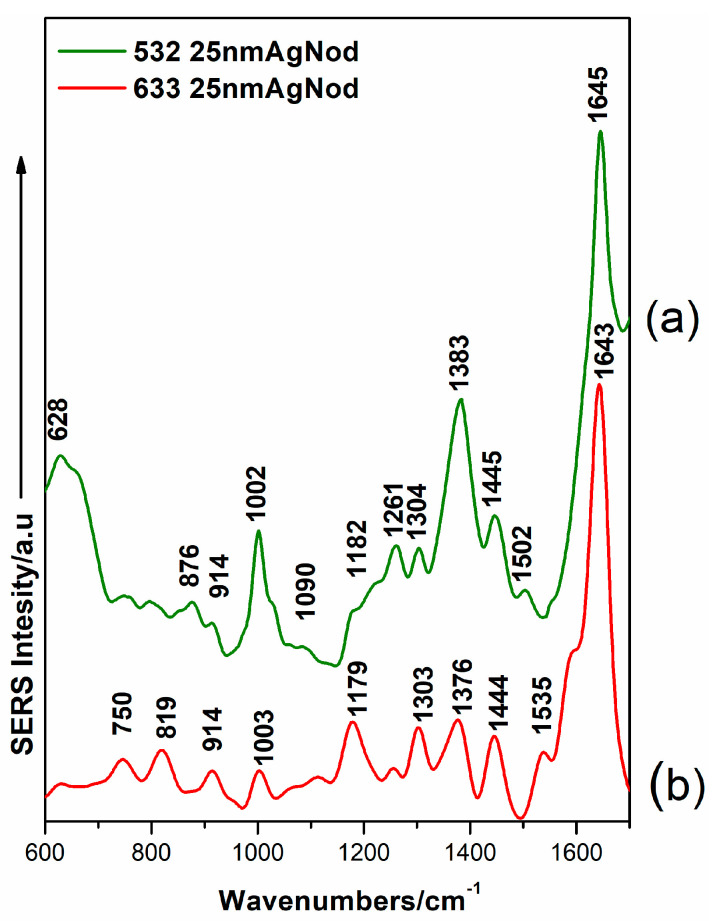
SERS spectra on of NOD/ethanol samples at 10^−3^ M concentration using the 532 nm laser line (**a**) and 633 nm laser line (**b**) using 25 nm Ag covered nanotrenches on plastic.

Figure 6 shows the SERS spectra of NOD obtained on a network of periodical nanotrenches covered with 25 nm-thick Ag layer, used as an enhancing substrate. It is worth mentioning that this design fabricated by NIL has shown promising results and an enhancement factor up to 10^7^ for crystal violet [81].

In our experimental attempts for NOD detection, we were able to assess the SERS profiles for a concentration of 10^-3^ M. However, these spectral features, shown for excitation with the 532 nm laser line (a) and 633 nm laser line (b), respectively, complement our previous experiments. The key marker bands for NOD detected in this case were more well-defined and had a higher signal-to-noise ratio. Table 2 shows the main marker Raman, the detection approaches used, and also relevant results from the existing literature on this topic. It includes the main SERS bands for NOD, detected by using several SERS platforms along with their performance for NOD trace level detection.

The specific Raman marker bands of NOD, detected by using DCDR technique are found at 1003 cm^−1^ and 1646 cm^−1^, respectively, and correspond to the phenyl-ring deformation and to the ν(C=C) vibrations [54]. These are the most intense bands detected by using both commercial substrates. Considering these two marker bands in SERS sensing also, NOD was detected using several SERS platforms (solid substrates and colloidal systems) with limits of detection found in the 10^−3^–10^−8^ M range.

## 3. Materials and Methods

### 3.1. Drop-Coating Deposition Raman Analysis of NOD

For Raman analysis, an ampule of 100 µg NOD (Cayman Chemical, Ann Arbor, MI, USA) was dissolved in 100 µL ethanol (Nordic Chemicals, Cluj-Napoca, Romania) as starting solution and several diluted concentrations were prepared, from 1.212 × 10^−3^ M to 1.121 × 10^−7^ M. The DCD process of 5–25 µL generated solid samples recrystallized as micro-deposits on a hydrophobic substrate (Tienta^®^ SpectRIM^TM^, EXW BioTools, Inc., Jupiter, FL, USA), which were further used for micro-Raman spectroscopy until the solvent evaporated.

Raman spectra were recorded using a Renishaw InVia Reflex Raman confocal spectrometer (Renishaw New Mills Wotton-under-Edge, Gloucestershire, UK) equipped with excitation lines found at 532 nm (200 mW), 633 nm (17 mW) and 785 nm (300 mW), respectively. The signal was collected from solid samples, recrystallized from a solution of 10^−3^ M NOD (by solving 100 µg in 100 µL of ethanol), in the range 100–3200 cm^−1^ by using a filter with edge > 100 cm^−1^ with spectral resolution of 1 cm^−1^. A Leica microscope equipped with a 20× objective was used to focus on and to visualize the samples. The Raman measurements were performed as follows: 40 s acquisitions at 5% laser power (532 nm); 30s acquisitions at 100% laser power (633 nm); and 50s acquisitions at 10% laser power (785 nm, 300 mW), respectively. We subtracted the baseline before plotting the acquired spectra in Origin 7.1 software (OriginLab Corporation, Northampton, MA, USA).

### 3.2. SERS Measurements 

#### 3.2.1. SERS Analysis on Klarite™ Substrates

For the NOD SERS measurements on solid substrate, we used a commercially available product, Klarite™, purchased from Mesophotonics Ltd. (Southampton Hampshire, UK), containing a silicon surface with inverted square pyramids covered with a gold layer [82]. These were calculated and fabricated to be able to produce localized surface plasmons, rendering SERS enhancement. Its high sensitivity and accuracy makes Klarite™ an affordable tool for use as a large-scale SERS analytical platform. For SERS analysis, NOD samples were analyzed using the Renishaw InVia Reflex Raman system aforementioned. Spectra were collected with the following experimental parameters: integration time 1 s; 1% laser power at 532 nm laser line.

#### 3.2.2. SERS on AgNPs

For the SERS analysis of NOD, AgNPs were prepared according to the classical, citrate-reduced procedure, as previously described [83]. In brief, 100 mL of aqueous solution containing 17 mg of AgNO_3_ salt (Sigma Aldrich, Merck KGaA, Darmstadt, Germany) was heated to boiling (100 °C) and then 2 mL of 1% trisodium citrate solution (Sigma Aldrich, Merck KGaA, Darmstadt, Germany) was added in drops, with constant stirring. The mixture was boiled for 45 min and left to cool down at room conditions. The freshly prepared colloidal AgNP solution was milky-grey in color, and exhibited an UV-VIS-absorption maximum at 424 nm and a featureless Raman spectrum. As such, prepared colloidal AgNPs according to this method have a size distribution centered around 40 nm [83]. The preparation of the SERS stock solutions follows the same steps—20 µL of different concentrations ranging from 10^−3^ to 10^−7^ M of NOD previously dissolved in ethanol was immersed in 400 µL AgNPs starting from a concentration of 10^−5^ M down to 10^−8^ M. For SERS analysis, 60 s exposure time and 5 acquisition at 10% laser power were set for each spectral acquisition.

#### 3.2.3. SERS on Substrates with Periodical Nanotrenches

The plastic substrate containing a periodical network of nanotrenches and nanogaps used as SERS substrate was fabricated using nanoimprint lithography (NIL) technique as previously reported [81]. Flexible IPS^®^-based polymeric substrates with a thickness of 500 μm were purchased from Obducat AB (Lund, Sweden). The custom-made 4.5 cm × 4.5 cm silicon (Si) mold (NIL Technologies, ApS, Kongens Lyngby, Denmark) containing a square area of periodic nanotrenches with a height of 300 nm and a pitch of 800 nm was fabricated using e-beam lithographic technique with lateral and vertical tolerances of +/− 15%. To prevent sticking, the Si mold was treated by the manufacturer with an antiadhesive layer of perfluorodecyltrichlorosilane. The periodic arrays of Ag nanotrenches and nanogaps were fabricated by thermal imprinting using a NIL Obducat EITRE^®^3 equipment (Obducat AB, Lund, Sweden). A maximum imprinting temperature of 155 °C and pressure of 40 bars were attained during the imprinting process. 

After the successful transfer of the nanopatterned area into the IPS^®^, a 25 nm Ag film was deposited at room temperature using Q150R PLUS sputtering coater equipment (Quorum Technologies Ltd., Lewes, UK) from a disk-style Ag target (57 mm diameter, 0.1 mm thickness) at a fixed distance of 27 mm from the substrate to source and a rotation rate of 5 rpm of the substrate. A base pressure of 10^−3^ mbar, a 35 mA sputter current using a DC power supply, and a deposition rate of 4 to 5 nm/min were kept constant during the Ag deposition. Scanning electron microscopy (SEM) characterization of the Ag substrate was presented in previous work [81].

The UV-Vis absorption spectrum of the substrate, which contains periodic nanotrenches, has two peaks at 318 nm and 363 nm as previously described [81]. Our previous study on 3D Ag-metallized nanotrenches revealed that a 25 nm silver-film covering NIL-imprinted nanopatterned IPS^®^ substrate is the most promising SERS-active platform [81] and therefore we chose it to detect NOD molecules at lower concentrations.

For SERS analysis of NOD, the DCD technique was applied for a 5 μL droplet of NOD solution at 10^−3^ M concentration. After deposition on the nanopatterned substrates, the NOD samples were analyzed using the Renishaw InVia Reflex Raman system aforementioned. Spectra were collected with the following experimental parameters: integration time 60 s; 10% laser power for 633 nm laser line and 20 s; 10% laser power for 532 nm laser line. A Leica microscope equipped with 100× and 20× objectives was used to focus and to visualize the sample.

### 3.3. Computational Details

Density functional theory (DFT) methods implemented in the Gaussian 16, revision C.01 [84] software package have been used for geometry optimizations and Raman spectra calculations of NOD. After careful investigation of the potential energy surface of the molecule in gas phase, five unique conformers have been identified. The most stable one, with the relative Boltzmann population of 68%, has been selected for Raman calculations (all the other conformers have Boltzmann populations less than 10%). 

The Austin–Frisch–Petersson functional including dispersion (APFD) [85] was used together with the 6-311+G(2d,p) triple-zeta basis set. The geometry optimization was set to meet tight criteria, while very tight criteria were imposed on the wavefunction convergence. The grid was set to ultrafine. Frequency calculations were performed at harmonic level. No imaginary frequencies were obtained, which indicates that the resulting geometry is a true minimum. All theoretical wavenumbers greater or equal to 1000 cm^−1^ include the 0.9621 scaling factor. 

The GaussView 6.1.1 software package [86] was used for output data analysis.

**Figure 7:**
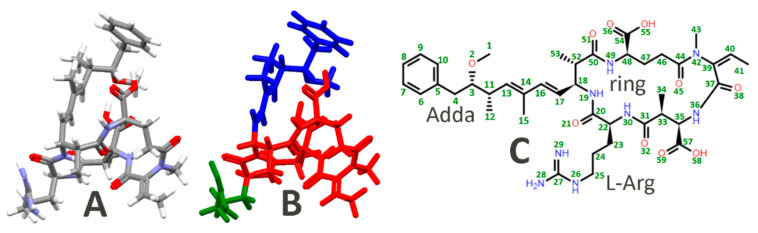
Optimized structure of NOD in gas phase at APFD/6-311+G(2d,p) level of theory (**A**) with the three main chemical components marked (**B**) in red—the main ring, blue—Adda, and green—L-Arg. The atom labels used in DFT calculations are shown on Nodularin’s chemical structure (**C**).

The calculated Raman activities S_i_ were converted to relative Raman intensities I_i_ by using Equation (1),

(1) $$I_{i}=\frac{f{\left(\nu_{0}-\nu_{i}\right)}^{4}S_{i}}{\nu_{i}\left(1-e^{-\frac{{\mathrm{hc}\nu }_{i}}{\mathrm{kT}}}\right)}$$

where: ν_0_—the excitation laser wavenumber (633 nm in this case); ν_i_—the wavenumber of the i^th^ normal mode; c—speed of light; h—Planck’s constant; k—Boltzmann’s constant; and T—temperature (293 K in this case). Both geometry optimization and frequency calculations were performed in gas phase, and the corresponding labels used for the chemical structure of NOD can be seen in Figure 7C.

## 4. Conclusions

Relying on theoretical DFT calculations, the fingerprint Raman marker bands of nodularin have been correctly assigned and further used to examine various label-free detection schemes for nodularin cyanotoxin. The results are important for further developing applications relying on Raman techniques for tracking cyanotoxin in environmental media. DCDR analysis was assessed for NOD and a LOD of 10^−4^ M was reached. SERS analysis was performed for NOD detection even at the 8.4 × 10^−8^ M level in colloidal AgNPs. We correlated SERS marker bands in terms of intensity and position with the DCDR specific signal of NOD obtained at higher concentration. Overall, both Raman and SERS analyses are prominent for further developing fast and effective detection schemes relying on Raman techniques. By using different laser lines as excitation sources, we were able to detect NOD with slightly shifted marker bands. The molecular orientation of NOD on different substrates with different plasmonic resonances is obviously different, according to the differences observed in the relative intensities of certain bands. Since the main Raman marker band at 1646 cm^−1^ is assigned to the skeletal stretching mode involving CC bonds, which is common for many microscystins, it is thus not specific for SERS detection of NOD in mixed toxins solution. It appeared that higher specificity was reached (due to the specific interaction) with the Klarite^TM^ and AgNPs nanoparticles. Nanotrenches apparently provided completely different orientations, with the highest enhancement of the skeletal stretching mode. The 532 nm excitation source provided more well-defined bands even at LOD levels, as well as the best performance in terms of intensity. This is reflected by the results obtained with Klarite^TM^ substrate and the silver-based colloidal system, situations that revealed the most promise for detection approaches and the lowest limits of detection.

## Figures and Tables

**Table 1:** Assignments of NOD from DFT-calculated Raman spectrum in gas phase, at APFD/6-311+G(2d,p) level of theory.

Raman 633 nm	APFD/ 6-311+G(2d,p)	Assignments
121 (s)	104	ρ(phenyl ring) + ρ(N28C27N29)
133	125	ρ(CH_2_) + γ(CH_3_) + γ(HN–C–NH)
	200	ρ(CH_3_)
234	220	ρ(CH_3_) + γ(HC–CH)
	261	γ(CH_3_) + δ(CH_2_–CH_2_–CH_2_) + γ(OCNH)
	277	γ(CH_3_) + γ(HC = CH)
	302	γ(OCNH)
	328	δ(H_3_C–CH–CH) + δ(HC–CH–COOH)
	351	δ(H_3_C–O–CH
621 (vw)	647	β(CCC) in phenyl ring–in plane def. of phenyl ring + γ(O55H)
737 (vw)	739	γ(N30H) + ω(C34H_3_)
823 (vw)	818	ν(C3C11) + ρ(C4H_2_) + ω(C12H_3_)
	848	ν(C4C5) + β(CCC) in phenyl ring–in plane def. of phenyl ring
883 (vw)	900	ν(C47C48) + ρ(C46H_2_) + ρ(C47H_2_) + β(C47C48H)
1003 (s)	985	β(CCC) on phenyl ring–in plane def. of phenyl ring
	1003	ω(CH_3_)
1028 (w)	1020	ω(CCC) + δ(CH)–on phenyl ring–in plane def. of phenyl ring
1064 (vw)	1043	ν(C23C24) + ω(CH_2_)
	1053	ν(C46C47) + ν(C47C48) + ω(CH_3_) + ω(CH_2_)
1100 (vw)	1074	ν(C22C23) + ν(C24C25) + β(C27N29H) + τ(N28H_2_) + ν(C52C53) + ω(C53H_3_)
	1085	ω(CH_3_) + γ(OH)
	1089	ω(CH_3_) + γ(OH) + τ(C4H_2_) + δ(CH) on phenyl ring
1153 (vw)	1157	ω(C1H_3_) + τ(C4H_2_) + δ(CH) on phenyl ring
1208 (vw)	1193	δ(CH) + δ(NH) + τ(CH_2_)
1229 (vw)	1223	δ(C11H) + δ(C13H) + δ(C14H) + δ(C16H) + ν(C11C13)
1257 (vw)	1246	ν(C57O58) + β(C57O58H)
1304 (vw)	1263	ν(C33C35) + ν(C35N36) + δ(C33H) + δ(C35H) + δ(N36H) + δ(C40H)
	1264	ν(C16C17) + δ(C16H) + δ(C17H) + δ(C52H)
1375 (vw)	1353	β_ip_(C41H_3_) + δ(C40H) + β_ip_(C43H_3_)
1452 (vw)	1419	β_oop_(C41H_3_) + β(C46H_2_)
1510 (vw)	1495	ν(37N36) + δ(N36H)
1581 (vw)	1597	ν(CC) in phenyl ring
1605 (w)	1634	ν(C37=O38) + ν(C39=C40) + β(C37N36H)
1645 (vs)	1664	ν(C13=C14) + ν(C16=C17)
	1681	ν(C39=C40) + ν(C37=O38) + ν(C57=C59)
	1700	ν(C57=O59) + β(C57O58H) + ν(C31=O32) + β(C31N30H) + β(C31C33H)
	1732	ν(C54=O56) + β(C54O55H) + β(C54C48H)
2880 (vw)	2889	ν_sym_(C25H_2_) + ν_sym_(C24H_2_)
	2901	ν_sym_(C1H_3_) + ν(C35H)
2922 (m)	2917	ν_sym_(C14H_3_)
2935 (m)	2933	ν_sym_(C12H_3_) + ν_sym_(C34H_3_)
	2957	ν(C18H)
	3001	ν(C4H_2_) + ν_as_(C12H_3_) + ν_as_(O58H) + ν_as_(C34H_3_) + ν(C42H_2_)
	3023	ν(O58H) + ν_as_(C1H_3_) + ν_as_(C12H_3_) + ν_as_(C15H_3_)
3060 (vw)	3084	ν(CH) on phenyl ring

Notes: ν—stretching; β—bending; δ—in plane bending; γ—out of plane bending; τ—twisting; ω—wagging; sym—symmetric; as—asymmetric; oop—out of phase; ip—in phase; vs.—very strong; s—strong; m—medium; w—weak; vw—very weak.

**Table 2:** Relevant Raman marker bands detected by using DCDR technique compared to those reported in [54] and SERS marker bands recorded by using the SERS platforms Ag sol, Ag nanotranches and Klarite^TM^. The limits of detection (LOD) for all three nanoplatforms are also indicated. NOD marker bands are highlighted in blue.

DCDR [54]	DCDR Technique/ Tienta SpectRIM^TM^ 532/633 nm LOD = 10^−4^ M	Ag Sol LOD = 8.4 × 10^−8^ M	Ag Nanotrenches LOD = 10^−3^ M	Klarite^TM^ LOD = 10^−7^ M
	621 m	612 m	621 m	671 w
752 w	736 w	775 m	750 m	727 w
834 m	822 m/822 m		819 m	
888 m	883 m/880 m		914 m	936 m
1006 s	1003 s/1002 s		1002 s/1003 s	
	1029 m			
1069 m	1064 w			1055 s
			1090 m	
			1179 m	
			1182 m	
1212 m	1207 m/1208 m			1205 s
1259 m	1256 m/1254 m			
1309 s	1304 s/1308 s		1303 m	1311 m
1379 m	1375m/1380 m	1362 s	1387 s	1368 m
1453 m	1452 s/1446 m	1452 s	1453 s	1415 m
		1509 m		
	1536/1582 w		1535 w/1553 w	1583 vs
1648 s	1645 s	1647 s	1645 s	1656 s

Notes: w—weak intensity; m—medium intensity; s—strong intensity; vs.—very strong.

## References

[1] Zahra Z., Choo D., Lee H., Parveen A. (2020). Cyanobacteria: Review of Current Potentials and Applications. Environments.

[2] Rasmussen B., Fletcher I.R., Brocks J.J., Kilburn M.R. (2008). Reassessing the first appearance of eukaryotes and cyanobacteria. Nature.

[3] Paerl H.W., Fulton R.S., Moisander P.H., Dyble J. (2001). Harmful freshwater algal blooms, with an emphasis on cyanobacteria. Sci. World J..

[4] Huisman J., Codd G.A., Paerl H.W., Ibelings B.W., Verspagen J.M.H., Visser P.M. (2018). Cyanobacterial blooms. Nat. Rev. Microbiol..

[5] Merel S., Walker D., Chicana R., Snyder S., Baures E., Thomas O. (2013). State of knowledge and concerns on cyanobacterial blooms and cyanotoxins. Environ. Int..

[6] Cazenave J., Wunderlin D.A., de Los Angeles Bistoni M., Ame M.V., Krause E., Pflugmacher S., Wiegand C. (2005). Uptake, tissue distribution and accumulation of microcystin-RR in *Corydoras paleatus*, *Jenynsia multidentata* and *Odontesthes bonariensis*. A field and laboratory study. Aquat. Toxicol..

[7] Carmichael W.W. (2001). Health Effects of Toxin-Producing Cyanobacteria: “The CyanoHABs”. Hum. Ecol. Risk Assess..

[8] Plaas H.E., Paerl H.W. (2021). Toxic Cyanobacteria: A Growing Threat to Water and Air Quality. Environ. Sci. Technol..

[9] Romanis C.S., Pearson L.A., Neilan B.A. (2021). Cyanobacterial blooms in wastewater treatment facilities: Significance and emerging monitoring strategies. J. Microbiol. Methods.

[10] Munoz M., Cires S., de Pedro Z.M., Colina J.A., Velasquez-Figueroa Y., Carmona-Jimenez J., Caro-Borrero A., Salazar A., Santa Maria Fuster M.C., Contreras D. (2021). Overview of toxic cyanobacteria and cyanotoxins in Ibero-American freshwaters: Challenges for risk management and opportunities for removal by advanced technologies. Sci. Total Environ..

[11] Duy T.N., Lam P.K., Shaw G.R., Connell D.W. (2000). Toxicology and risk assessment of freshwater cyanobacterial (blue-green algal) toxins in water. Rev. Environ. Contam. Toxicol..

[12] Chorus I., Falconer I.R., Salas H.J., Bartram J. (2000). Health risks caused by freshwater cyanobacteria in recreational waters. J. Toxicol. Environ. Health B Crit. Rev..

[13] Chen L., Chen J., Zhang X., Xie P. (2016). A review of reproductive toxicity of microcystins. J. Hazard. Mater..

[14] Humpage A. (2008). Toxin types, toxicokinetics and toxicodynamics. Adv. Exp. Med. Biol..

[15] Ibelings B.W., Chorus I. (2007). Accumulation of cyanobacterial toxins in freshwater “seafood” and its consequences for public health: A review. Environ. Pollut..

[16] Dietrich D., Hoeger S. (2005). Guidance values for microcystins in water and cyanobacterial supplement products (blue-green algal supplements): A reasonable or misguided approach?. Toxicol. Appl. Pharmacol..

[17] Lage S., Mazur-Marzec H., Gorokhova E. (2021). Competitive interactions as a mechanism for chemical diversity maintenance in *Nodularia spumigena*. Sci. Rep..

[18] Gehringer M.M., Adler L., Roberts A.A., Moffitt M.C., Mihali T.K., Mills T.J., Fieker C., Neilan B.A. (2012). Nodularin, a cyanobacterial toxin, is synthesized in planta by symbiotic *Nostoc* sp.. ISME J..

[19] Moffitt M.C., Blackburn S.I., Neilan B.A. (2001). rRNA sequences reflect the ecophysiology and define the toxic cyanobacteria of the genus Nodularia. Int. J. Syst. Evol. Microbiol..

[20] Beattie K.A., Kaya K., Codd G.A. (2000). The cyanobacterium *Nodularia* PCC 7804, of freshwater origin, produces [L-Har^2^]nodularin. Phytochemistry.

[21] Dittmann E., Wiegand C. (2006). Cyanobacterial toxins—Occurrence, biosynthesis and impact on human affairs. Mol. Nutr. Food Res..

[22] Jokela J., Heinila L.M.P., Shishido T.K., Wahlsten M., Fewer D.P., Fiore M.F., Wang H., Haapaniemi E., Permi P., Sivonen K. (2017). Production of High Amounts of Hepatotoxin Nodularin and New Protease Inhibitors Pseudospumigins by the Brazilian Benthic *Nostoc* sp. CENA543. Front. Microbiol..

[23] Meissner S., Fastner J., Dittmann E. (2013). Microcystin production revisited: Conjugate formation makes a major contribution. Environ. Microbiol..

[24] Codd G.A., Morrison L.F., Metcalf J.S. (2005). Cyanobacterial toxins: Risk management for health protection. Toxicol. Appl. Pharmacol..

[25] Massey I.Y., Wu P., Wei J., Luo J., Ding P., Wei H., Yang F. (2020). A Mini-Review on Detection Methods of Microcystins. Toxins.

[26] Nagata S., Tsutsumi T., Hasegawa A., Yoshida F., Ueno Y., Watanabe M.F. (2020). Enzyme Immunoassay for Direct Determination of Microcystins in Environmental Water. J. AOAC Int..

[27] Agrawal M., Yadav S., Patel C., Raipuria N. (2012). Bioassay methods to identify the presence of cyanotoxins in drinking water supplies and their removal strategies. Eur. J. Exp. Biol..

[28] Forastier M.E., Zalocar Y., Andrinolo D., Domitrovic H.A. (2016). Occurrence and toxicity of *Microcystis aeruginosa* (Cyanobacteria) in the Parana River, downstream of the Yacyreta dam (Argentina). Rev. Biol. Trop..

[29] Vasconcelos V.M., Sivonen K., Evans W.R., Carmichael W.W., Namikoshi M. (1996). Hepatotoxic microcystin diversity in cyanobacterial blooms collected in portuguese freshwaters. Water Res..

[30] McLellan N.L., Manderville R.A. (2017). Toxic mechanisms of microcystins in mammals. Toxicol. Res..

[31] Rastogi R.P., Sinha R.P., Incharoensakdi A. (2014). The cyanotoxin-microcystins: Current overview. Rev. Environ. Sci. Biotechnol..

[32] MacKintosh C., Beattie K.A., Klumpp S., Cohen P., Codd G.A. (1990). Cyanobacterial microcystin-LR is a potent and specific inhibitor of protein phosphatases 1 and 2A from both mammals and higher plants. FEBS Lett..

[33] Ikehara T., Kuniyoshi K., Yamaguchi H., Tanabe Y., Sano T., Yoshimoto M., Oshiro N., Nakashima S., Yasumoto-Hirose M. (2019). First Report of Microcystis Strains Producing MC-FR and -WR Toxins in Japan. Toxins.

[34] Watson S.B., Zastepa A., Boyer G.L., Matthews E. (2017). Algal bloom response and risk management: On-site response tools. Toxicon.

[35] Nasri A.B., Bouaicha N., Fastner J. (2004). First report of a microcystin-containing bloom of the cyanobacteria *Microcystis* spp. in Lake Oubeira, eastern Algeria. Arch. Environ. Contam. Toxicol..

[36] Wharton R.E., Cunningham B.R., Schaefer A.M., Guldberg S.M., Hamelin E.I., Johnson R.C. (2019). Measurement of Microcystin and Nodularin Activity in Human Urine by Immunocapture-Protein Phosphatase 2A Assay. Toxins.

[37] Kleinteich J., Puddick J., Wood S.A., Hildebrand F., Laughinghouse H.I., Pearce D.A., Dietrich D.R., Wilmotte A. (2018). Toxic Cyanobacteria in Svalbard: Chemical Diversity of Microcystins Detected Using a Liquid Chromatography Mass Spectrometry Precursor Ion Screening Method. Toxins.

[38] Botha C.J., Laver P.N., Singo A., Venter E.A., Ferreira G.C.H., Rösemann M., Myburgh J.G. (2018). Evaluation of a Norwegian-developed ELISA to determine microcystin concentrations in fresh water. Water Supply.

[39] Baralla E., Varoni M.V., Sedda T., Pasciu V., Floris A., Demontis M.P. (2017). Microcystins Presence in Mussels (*M. galloprovincialis*) and Water of Two Productive Mediterranean’s Lagoons (Sardinia, Italy). Biomed. Res. Int..

[40] Geis-Asteggiante L., Lehotay S.J., Fortis L.L., Paoli G., Wijey C., Heinzen H. (2011). Development and validation of a rapid method for microcystins in fish and comparing LC-MS/MS results with ELISA. Anal. Bioanal. Chem..

[41] Müller C., Glamuzina B., Pozniak I., Weber K., Cialla D., Popp J., Cîntă-Pînzaru S. (2014). Amnesic shellfish poisoning biotoxin detection in seawater using pure or amino-functionalized Ag nanoparticles and SERS. Talanta.

[42] Cîntă-Pînzaru S., Müller C., Todor I.S., Glamuzina B., Chiș V. (2016). NIR-Raman spectrum and DFT calculations of okadaic acid DSP marine biotoxin microprobe. J. Raman Spectrosc..

[43] Cîntă-Pînzaru S., Müller C., Ujevic I., Vențer M.M., Chiș V., Glamuzina B. (2018). Lipophilic marine biotoxins SERS sensing in solutions and in mussel tissue. Talanta.

[44] Sipia V.O., Karlsson K.M., Meriluoto J.A., Kankaanpaa H.T. (2004). Eiders (*Somateria mollissima*) obtain nodularin, a cyanobacterial hepatotoxin, in Baltic Sea food web. Environ. Toxicol. Chem..

[45] Kankaanpaa H., Turunen A.K., Karlsson K., Bylund G., Meriluoto J., Sipia V. (2005). Heterogeneity of nodularin bioaccumulation in northern Baltic Sea flounders in 2002. Chemosphere.

[46] Sipia V.O., Sjovall O., Valtonen T., Barnaby D.L., Codd G.A., Metcalf J.S., Kilpi M., Mustonen O., Meriluoto J.A. (2006). Analysis of nodularin-R in eider (*Somateria mollissima*), roach (*Rutilus rutilus* L.), and flounder (*Platichthys flesus* L.) liver and muscle samples from the western Gulf of Finland, northern Baltic Sea. Environ. Toxicol. Chem..

[47] Stewart I., Eaglesham G.K., McGregor G.B., Chong R., Seawright A.A., Wickramasinghe W.A., Sadler R., Hunt L., Graham G. (2012). First Report of a Toxic *Nodularia spumigena* (Nostocales/Cyanobacteria) Bloom in Sub-Tropical Australia. II. Bioaccumulation of Nodularin in Isolated Populations of Mullet (Mugilidae). Int. J. Environ. Res. Public Health.

[48] Zhang H.Y., Yang S.P., Beier R.C., Beloglazova N.V., Lei H.T., Sun X.L., Ke Y.B., Zhang S.X., Wang Z.H. (2017). Simple, high efficiency detection of microcystins and nodularin-R in water by fluorescence polarization immunoassay. Anal. Chim. Acta.

[49] Ouyang S.Q., Hu B., Zhou R., Liu D.J., Peng D.F., Li Z.G., Li Z., Jiao B.H., Wang L.H. (2018). Rapid and sensitive detection of nodularin-R in water by a label-free BLI aptasensor. Analyst.

[50] Akter S., Kustila T., Leivo J., Muralitharan G., Vehniainen M., Lamminmaki U. (2019). Noncompetitive Chromogenic Lateral-Flow Immunoassay for Simultaneous Detection of Microcystins and Nodularin. Biosensors.

[51] Wang L., Liu W., Tang J.W., Wang J.J., Liu Q.H., Wen P.B., Wang M.M., Pan Y.C., Gu B., Zhang X. (2021). Applications of Raman Spectroscopy in Bacterial Infections: Principles, Advantages, and Shortcomings. Front. Microbiol..

[52] Koya S.K., Yurgelevic S., Brusatori M., Huang C., Diebel L.N., Auner G.W. (2019). Rapid Detection of *Clostridium difficile* Toxins in Stool by Raman Spectroscopy. J. Surg. Res..

[53] Halvorson R.A., Leng W., Vikesland P.J. (2011). Differentiation of Microcystin, Nodularin, and Their Component Amino Acids by Drop-Coating Deposition Raman Spectroscopy. Anal. Chem..

[54] Halvorson R.A., Vikesland P.J. (2011). Drop Coating Deposition Raman (DCDR) for Microcystin-LR Identification and Quantitation. Environ. Sci. Technol..

[55] Müller Molnár C., Cîntă Pînzaru S., Chiș V., Feher I., Glamuzina B. (2023). SERS of cylindrospermopsin cyanotoxin: Prospects for quantitative analysis in solution and in fish tissue. Spectrochim. Acta Part A.

[56] Pang P., Lai Y., Zhang Y., Wang H., Conlan X.A., Barrow C.J., Yang W. (2020). Recent Advancement of Biosensor Technology for the Detection of Microcystin-LR. Bull. Chem. Soc. Jpn..

[57] Li M., Paidi S.K., Sakowski E., Preheim S., Barman I. (2019). Ultrasensitive Detection of Hepatotoxic Microcystin Production from Cyanobacteria Using Surface-Enhanced Raman Scattering Immunosensor. ACS Sensors.

[58] Zong C., Xu M., Xu L.-J., Wei T., Ma X., Zheng X.-S., Hu R., Ren B. (2018). Surface-Enhanced Raman Spectroscopy for Bioanalysis: Reliability and Challenges. Chem. Rev..

[59] Lane L.A., Qian X., Nie S. (2015). SERS Nanoparticles in Medicine: From Label-Free Detection to Spectroscopic Tagging. Chem. Rev..

[60] Schlücker S. (2014). Surface-Enhanced Raman Spectroscopy: Concepts and Chemical Applications. Angew. Chem. Int. Ed..

[61] Wang Y., Yan B., Chen L. (2013). SERS Tags: Novel Optical Nanoprobes for Bioanalysis. Chem. Rev..

[62] Chao J., Cao W., Su S., Weng L., Song S., Fan C., Wang L. (2016). Nanostructure-based surface-enhanced Raman scattering biosensors for nucleic acids and proteins. J. Mater. Chem. B.

[63] Zhao Y., Yang X., Li H., Luo Y., Yu R., Zhang L., Yang Y., Song Q. (2015). Au nanoflower–Ag nanoparticle assembled SERS-active substrates for sensitive MC-LR detection. Chem. Commun..

[64] Hassanain W.A., Izake E.L., Schmidt M.S., Ayoko G.A. (2017). Gold nanomaterials for the selective capturing and SERS diagnosis of toxins in aqueous and biological fluids. Biosens. Bioelectron..

[65] He S., Xie W., Fang S., Zhou D., Djebbi K., Zhang Z., Du J., Du C., Wang D. (2019). Label-free identification of trace microcystin-LR with surface-enhanced Raman scattering spectra. Talanta.

[66] Cîntă-Pînzaru S., Müller C., Tomšić S., Vențer M.M., Brezeștean I., Ljubimir S., Glamuzina B. (2016). Live diatoms facing Ag nanoparticles: Surface enhanced Raman scattering of bulk *Cylindrotheca closterium* pennate diatoms and of the single cells. RSC Adv..

[67] Li M., Lin H., Paidi S.K., Mesyngier N., Preheim S., Barman I. (2020). A Fluorescence and Surface-Enhanced Raman Spectroscopic Dual-Modal Aptasensor for Sensitive Detection of Cyanotoxins. ACS Sens..

[68] Luo X., Zhao X., Wallace G.Q., Brunet M.-H., Wilkinson K.J., Wu P., Cai C., Bazuin C.G., Masson J.-F. (2021). Multiplexed SERS Detection of Microcystins with Aptamer-Driven Core–Satellite Assemblies. ACS Appl. Mater. Interfaces.

[69] Bostan H.B., Taghdisi S.M., Bowen J.L., Demertzis N., Rezaee R., Panahi Y., Tsatsakis A.M., Karimi G. (2018). Determination of microcystin-LR, employing aptasensors. Biosens. Bioelectron..

[70] Zhang D., Xie Y., Mrozek M.F., Ortiz C., Davisson V.J., Ben-Amotz D. (2003). Raman detection of proteomic analytes. Anal. Chem..

[71] Zhang D., Mrozek M.F., Xie Y., Ben-Amotz D. (2004). Chemical Segregation and Reduction of Raman Background Interference Using Drop Coating Deposition. Appl. Spectrosc..

[72] Ortiz C., Zhang D., Xie Y., Ribbe A.E., Ben-Amotz D. (2006). Validation of the drop coating deposition Raman method for protein analysis. Anal. Biochem..

[73] Kočišová E., Sayedová S., Procházka M. (2020). Drop coating deposition Raman scattering of selected small molecules of biological importance. J. Raman Spectrosc..

[74] Kuizova A., Prikryl M., Prochazka M., Kočišová E. (2021). Drop coating deposition Raman (DCDR) spectroscopy of contaminants. Spectrochim. Acta Part A.

[75] Kočišová E., Procházka M., Vaculciakova L. (2015). Drop-Coating Deposition Raman (DCDR) Spectroscopy as a Tool for Membrane Interaction Studies: Liposome-Porphyrin Complex. Appl. Spectrosc..

[76] Dutta S.B., Krishna H., Khan K.M., Shrivastava R., Sahu K., Gupta S., Majumder S.K. (2020). Drop-coating deposition Raman spectroscopy for quantitative detection of urinary creatinine: A feasibility study. Laser Phys..

[77] Li P., Chen C., Deng X., Mao H., Jin S. (2015). Drop coating deposition Raman spectroscopy of blood plasma for the detection of colorectal cancer. J. Biomed. Opt..

[78] Tahir M.A., Zhang X., Cheng H., Xu D., Feng Y., Sui G., Fu H., Valev V.K., Zhang L., Chen J. (2020). Klarite as a label-free SERS-based assay: A promising approach for atmospheric bioaerosol detection. Analyst.

[79] Martina I., Wiesinger R., Jembrih-Simbuerger D., Schreiner M. (2012). Micro-Raman characterisation of silver corrosion products. e-PS.

[80] Gao X., Davies J.P., Weaver M.J. (1990). Test of surface selection rules for surface-enhanced Raman scattering: The orientation of adsorbed benzene and monosubstituted benzenes on gold. J. Phys. Chem..

[81] Colniță A., Marconi D., Dina N.E., Brezeștean I., Bogdan D., Turcu I. (2022). 3D silver metallized nanotrenches fabricated by nanoimprint lithography as flexible SERS detection platform. Spectrochim. Acta Part A.

[82] Zhang S., Fan W., Panoiu N.C., Malloy K.J., Osgood R.M., Brueck S.R.J. (2006). Optical negative-index bulk metamaterials consisting of 2D perforated metal-dielectric stacks. Opt. Express.

[83] Lee P.C., Meisel D. (1982). Adsorption and surface-enhanced Raman of dyes on silver and gold sols. J. Phys. Chem..

[84] Frisch M., Trucks G.W., Schlegel H.B., Scuseria G.E., Robb M.A., Cheeseman J.R., Scalmani G., Barone V., Mennucci B., Petersson G.A. (2013). Gaussian 09 Revision E.01.

[85] Austin A., Petersson G.A., Frisch M.J., Dobek F.J., Scalmani G., Throssell K. (2012). A Density Functional with Spherical Atom Dispersion Terms. J. Chem. Theory Comput..

[86] Dennington R., Keith T.A., Millam J.M. (2016). Gauss View.

